# Patient Satisfaction and Surgeon Experience: A Follow-Up to the Reduction Mammaplasty Learning Curve Study

**Published:** 2012-05-04

**Authors:** Matthew J. Carty, Antoine Duclos, Xiangmei Gu, Nkemdiche Elele, Dennis Orgill

**Affiliations:** ^a^Division of Plastic Surgery, Brigham and Women's Hospital/Faulkner Hospital, Jamaica Plain, MA; ^b^Brigham and Women's Hospital Center for Surgery and Public Health, Boston, MA; ^c^Harvard Medical School, Boston, MA

## Abstract

**Background:** While it is known that increasing surgeon experience is correlated with improved efficiency and safety in the reduction mammaplasty procedure, it is unclear whether these improvements lead to an erosion in patient satisfaction. **Methods:** The authors distributed the Breast-Q questionnaire to all patients who underwent bilateral reduction mammaplasty at their institution between 1995 and 2007. Univariate and multivariate analyses were performed to assess the relationship between postoperative patient satisfaction scores and surgeon experience, as well as to characterize those patients with particularly high or low satisfaction scores, in general. **Results:** A total of 279 (26.1%) completed surveys were analyzed. No statistically significant erosion in either Satisfaction with Breasts (SWB) or Satisfaction with Outcomes (SWO) scores were witnessed with increasing surgeon experience or efficiency. Patients older than 40 years demonstrated significantly higher SWB scores than younger patients (*P* = .004), while patients who suffered postoperative soft tissue necrosis demonstrated significantly lower SWB (*P* = .003) and SWO (*P* = .010) scores. **Conclusions:** Gains in operative efficiency with increasing surgeon experience do not appear to come at the expense of patient satisfaction in the reduction mammaplasty procedure. Younger patients and those who experience postoperative soft tissue necrosis appear to be at higher risk for reporting lower postoperative patient satisfaction scores.

Our prior investigation into the dynamics underlying the reduction mammaplasty procedural learning curve revealed the primacy of surgeon experience with regard to performance improvement.^1^ Consideration of our findings permitted us to postulate a theoretical model with 3 stages of evolution, characterized by improving surgical efficiency and declining complication rates with each successive phase. The results of this analysis have provided the foundation for an iterative, prospective approach to performance improvement, similar to that employed in other arenas of health care and in general industry; the development of this methodology is the focus of our research group.

In seeking to further refine this new approach to performance improvement, however, we have given serious consideration to the lack of metrics related to patient satisfaction and esthetic outcomes in our learning curve analysis. It is well-recognized that the reduction mammaplasty procedure serves both functional and esthetic aims, and that effective fulfillment of both priorities is vital to this intervention's success.^2^ Towards this end, we acknowledge that any thoughtful consideration of the reduction mammaplasty procedure must incorporate outcome measures that take account of cosmesis, in addition to efficiency and safety metrics, and have undertaken the present study in the interests of addressing this concern.

The purpose of this study was to supplement the findings of our prior investigation through a thoughtful consideration of patient satisfaction trends over the course of the reduction mammaplasty learning curve. In particular, our focus was to elucidate whether increasing surgeon efficiency over time resulted in a concomitant erosion in patient satisfaction. A secondary goal was to determine those factors most readily associated with improved or diminished patient satisfaction.

## METHODS

The generation of our core patient database has been described in detail elsewhere.^1^ In brief, we reviewed all female bilateral reduction mammaplasty procedures performed at Brigham and Women's Hospital between January 18, 1995, and December 31, 2007, by 8 attending surgeons. Data were culled from a combination of electronic medical records, an electronic operative time tracking application and physician employee databases. Breast-Q questionnaires (postoperative reduction module 1.0)^3^ were sent to all patients in the database with logged mailing addresses; these questionnaires were accompanied by a letter from the lead author detailing the intent of the study and inviting voluntary participation. The results of all returned questionnaires were tallied and converted into numeric values via the Q-Score application.^4^

Statistical analyses were then conducted using SAS 9.2 (SAS Institute Inc, Cary, NC), for which mean confidence intervals were set at 95% (CI 95%) and *P* values were 2-sided. To assess the representativeness of the population who completed the satisfaction survey, characteristics were compared between responders and nonresponders. Chi-squared or Fisher exact tests were used, as appropriate, to compare categorical variables, whereas the Student *t* test was employed to compare continuous variables.

Among the responders, nonparametric analyses were performed to identify variables potentially associated with scoring of 2 cardinal satisfaction domains: “Satisfaction with Breasts” (SWB) and “Satisfaction with Outcomes” (SWO). A Kruskal-Wallis test or Mann-Whitney Wilcoxon test was used to compare satisfaction scores between groups. Correlation between satisfaction scores and surgeon experience, as well as operative time, was also tested using the Spearman's rank correlation test. For every variable of interest, mean value or percentage was computed with corresponding 95% CI according to stratification on tertiles of SWB and SWO scores. Last, multivariate analyses for potential associations between these covariates and patient satisfaction were performed using generalized estimating equations, accounting for clustering of patients by surgeon.

## RESULTS

A total of 1068 breast-reduction procedures were performed during the study period; of these, 279 (26.1%) completed the satisfaction survey. No significant differences were observed between responder and nonresponder characteristics except for patient's age and the year of procedure; younger patients or those who underwent operation prior to 1998 completed the survey less frequently (Table 1).

Univariate analyses of survey responders demonstrated SWB and SWO mean scores of 67.6 [95% CI, 65.1-70.1] and 82.8 [95% CI, 80.4-85.1], respectively (Table 2). No significant differences in mean scores were observed between surgeons or by years of surgeon experience. Furthermore, no correlation was observed between experience and SWB (ρ = 0.09, *P* = .145) or SWO (ρ = 0.08, *P* = .176), nor between operative time and SWB (ρ = -0.07, *P* = .222) or SWO (ρ = -0.01, *P* = .840). Postoperative necrosis was the only factor associated with both SWB and SWO domains: the mean SWB score was lower among patients experiencing postoperative necrosis (50.2 [95% CI, 35.4-65.1]) compared with those who did not (68.1 [95% CI, 65.6-70.6]), with a similar reduction witnessed in mean SWO scores (64.4 [95% CI, 51.8-77.1] vs 83.3 [95% CI, 80.9-85.6]). Higher SWB scores were also linked with increasing patient age. These results were recapitulated in additional analyses of SWB and SWO scores stratified by quartiles; patients having high SWB scores were generally older than lower scoring patients (*P* = .004), and postoperative necrosis was never witnessed among patients with high SWB or SWO scores (*P* = .003, .010, respectively) (Table 3).

As in the univariate analyses, multivariate analyses were notable for a statistically significant association between increasing patient age and SWB. On the basis of composite outcomes, a nonsignificant trend was also observed between the occurrence of complications and SWO. No relationship was found between satisfaction scores and surgeon's experience or operative time, respectively (Table 4).

## DISCUSSION

Applying a uniform standard to the evaluation of breast esthetics—particularly in the postoperative period—is not a straightforward task. The plastic surgery literature includes attempts by multiple investigators to determine a universally acceptable, independent system for the assessment of breast cosmesis, including expositions on the potential virtues of subjective ratings scales,^2^ physical measurements,^5^ photographic measurements, and 3-dimensional imaging.^6^^,^^7^ To date, no consensus regarding the applicability of any single *surgeon-reported* outcomes measurement tool has been achieved.

Perhaps in response, attention has increasingly turned instead to the employment of *patient-reported* outcomes measurement tools to assess a variety of esthetic, functional, and psychological parameters related to breast procedures. A review of the literature demonstrates a veritable cornucopia of patient satisfaction questionnaires that have been applied to the assessment of breast surgery outcomes, in general, and reduction mammaplasty, in particular, including the Short Form 36,^8^^,^^9^ the Multidimensional Body-Self Relations Questionnaire,^10^ the Breast Evaluation Questionnaire,^11^ and the Brief Symptom Inventory,^12^ to name a few. Studies utilizing these questionnaires have consistently demonstrated improvement in health status and psychological well-being among patients following reduction mammaplasty, bolstering claims regarding the functional and emotional utility of this procedure.^15^^-^21

However, a major criticism of utilizing patient-reported outcomes measurement tools in the assessment of reduction mammaplasty patients is that these ad hoc questionnaires tend to lack validity, reliability, and specificity.^22^ Recognition of these limitations has prompted a recent interest in defining explicit guidelines for the development of procedure-specific, patient-reported outcomes measurement tools in plastic surgery^23^^,^^24^ and an enhanced appreciation for the value of such metrics; as noted by Clapham et al, “In contrast to using traditional outcome measures such as mortality and morbidity, plastic surgery is a quality-of-life specialty in which the satisfaction of the patient may be the most important outcomes metric in determining whether the patient will return for additional reconstructive or esthetic procedures.”25(p1826)

In accordance with these guidelines, Pusic et al^3^ introduced the BREAST-Q questionnaire in 2009 as a validated, reliable patient satisfaction assessment tool for reconstructive and esthetic breast surgery procedures. Developed through a rigorous process of conceptual framework formation, item generation, and psychometric evaluation, the BREAST-Q includes separate modules for the assessment of patients who have undergone cosmetic, reconstructive, and reduction breast surgery procedures. The breast reduction module, specifically, consists of 93 questions distributed across 10 broad categories of interest including SWB esthetics, satisfaction with overall outcome, psychosocial and sexual well-being, and satisfaction with surgeon/medical/office staff.^26^ Patient responses to questionnaires are tabulated and scored through the employment of the QSCORE application, which consolidates responses to single numeric value for each category ranging from 0 (*very dissatisfied*) to 100 (*very satisfied*).^4^

We selected the BREAST-Q as our analytic instrument because of its documented validity, reliability, and specificity for reduction mammaplasty patients. To our knowledge, our study represents the largest cohort of BREAST-Q responses for this procedure reported to date. We focused our analysis principally on 2 of the 10 possible satisfaction categories—SWB and SWO—in accordance with our stated intent to preferentially concentrate on the esthetic components of the procedure; our analyses therefore pertain to a subset of the patient-reported responses derived from our use of the BREAST-Q.

Our results suggest that patient assessments of esthetics and overall outcomes following reduction mammaplasty do not appear to decline with increasing operative efficiency. In fact, patient satisfaction in these 2 categories seems to exhibit essential stability over the course of the procedural learning curve, with some hint of slow but gradual ongoing improvement with increasing surgeon experience. This improvement may be attributable to declining complication rates witnessed in the later phases of the reduction mammaplasty learning curve, as evidenced in the association noted between postoperative skin necrosis and reduced satisfaction scores. Interestingly, this finding runs counter to that of other investigators such as Cunningham et al,^10^ who noted no relationship between complications and patient satisfaction scores in reduction mammaplasty. The link between increasing patient age and improved SWB may be due to a variety of causes, the most likely of which is, in our opinion, increasingly realistic expectations with advanced age.

**Figure 1:**
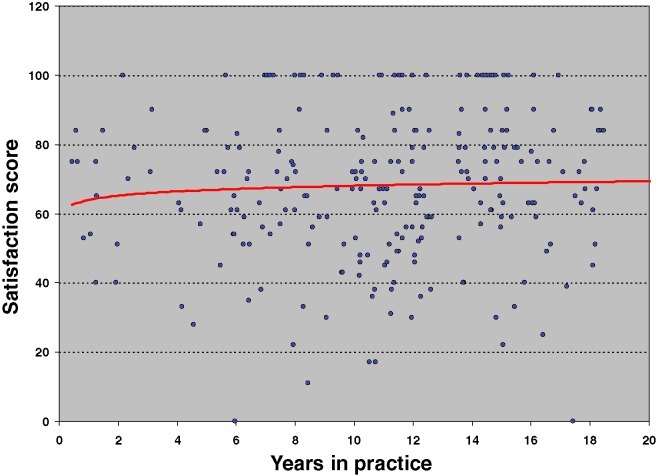
Satisfaction with breast esthetics versus surgeon experience.

**Figure 2:**
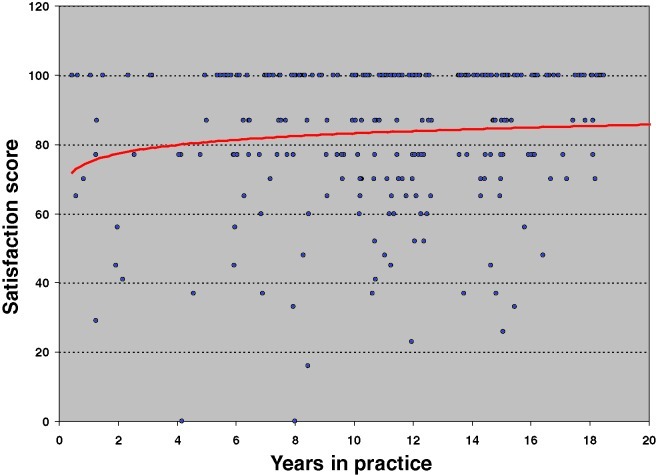
Satisfaction with overall outcome versus surgeon experience.

**Figure 3:**
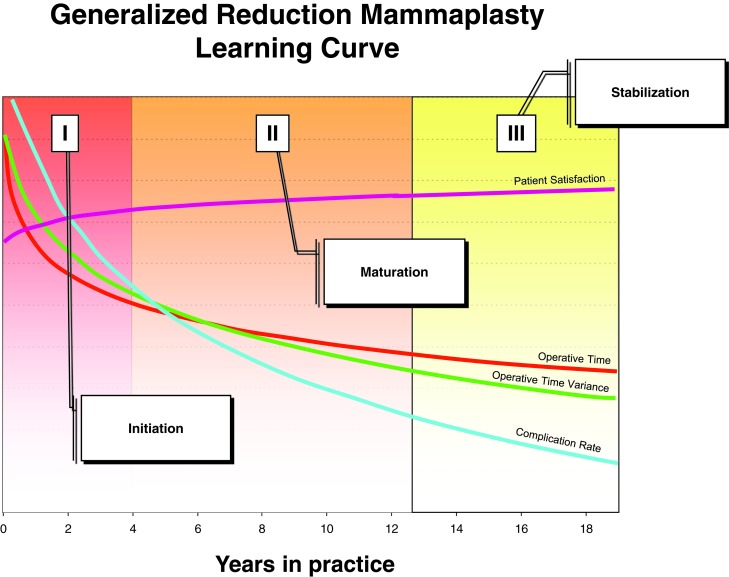
Updated operative learning curve conceptual model.

These findings support the hypothesis that efficiency gains achieved over the course of the reduction mammaplasty learning curve do not appear to come at the expense of patient satisfaction with regard to postoperative esthetics or overall outcome. In fact, the benefits realized though diminished operative time and operative time variance with increasing surgeon experience may be accompanied concomitantly not only by improved safety as reflected in diminishing complication rates but also by stable patient satisfaction scores with ongoing maturation of the operative surgeon (Figs 1 and 2). This salient finding permits us to propose an updated rendition of our reduction mammaplasty learning curve conceptual model that now incorporates not only efficiency and safety trends but also patient satisfaction trends, over the natural evolution of the procedure (Fig 3).

A curious finding of our study is that patient satisfaction scores appear to plateau very early for attending-level surgeons and remain largely constant over the course of the reduction mammaplasty learning curve. This contrasts starkly with the ongoing improvement witnessed in efficiency and safety outcomes over the same interval that we have previously reported. The reasons underlying this dynamic remain unclear; perhaps the development of social proficiency precedes the development of technical proficiency by many years, with the most marked gains in the former witnessed over the course of residency training (which is not included in our analyses). Why this evolution would occur over a different interval than that observed for efficiency and safety improvements is open to speculation and is a potentially fertile area for additional research.

While the primary intent of our investigation was to determine whether patient satisfaction erodes with increasing surgeon efficiency in reduction mammaplasty, a secondary goal of our study was to elucidate those factors that may contribute to either exceptionally high or exceptionally low patient satisfaction scores in this procedure, in general. Our analyses of patient satisfaction scores stratified by score quartile suggest that patient age appears to correlate with procedural satisfaction, with increased age (particularly >40 years) associated with increased satisfaction. As referenced previously, this may be a reflection of lower or more realistic expectations on the part of older patients; alternately, it may evidence that older patients have lived with the negative sequelae of macromastia for a longer duration and, therefore, find a greater degree of relief both functionally and esthetically after undergoing reduction mammaplasty. This finding suggests that additional care should be taken when setting postoperative expectations in the preoperative setting in patients younger than 40 years. In addition, lower satisfaction scores seem to be reported among patients who suffer postoperative complications; this finding is particularly evident among patients who experience postoperative skin necrosis—likely due to the fact that treatment in this population generally consists of prolonged conservative wound care with an increased likelihood of subsequent prominent scarring. This finding provides credence to the well-established notion of limiting tension along closure lines and counseling patients to avoid agents known to contribute to poor wound healing, such as tobacco exposure.

There are several limitations to the present study that warrant mention:

1. Our study represents a retrospective, cross-sectional survey, and therefore falls prey to the limitations generally witnessed in this study design. In addition, our lack of prospective data collection did not permit an assessment of respondents' preoperative satisfaction levels; we are therefore making an implicit assumption that all respondents demonstrated an approximately equivalent baseline level of satisfaction. Furthermore, despite the absence of marked differences between the responder and nonresponder subgroups, it is unclear whether those patients who completed questionnaires may exhibit either a positive or negative bias with regard to their outcomes. Last, the lack of longitudinal satisfaction tracking of individual patients does not permit us to comment on the durability of patient assessments over time. However, other published studies concerning long-term patient satisfaction and reduction mammaplasty suggest that satisfaction scores tend to improve steadily over the course of the first postoperative year, then stabilize and remain essentially constant thereafter.^27^^,^^28^
2. Our reliance on patient-reported measures for esthetic outcome assumes an implicit equivalency between these metrics and objective, third-party assessments commonly employed in other studies. This assumption, however, is not unfounded; several reports by other investigators have noted patient evaluations to be a reliable index of esthetic outcome following reduction mammaplasty, often identical in scale to evaluations provided by expert, objective observers.^29^^,^^30^
3. Our results are based on an assessment of dynamics witnessed in a multiple-member faculty practice in a large academic medical center. It is uncertain to what degree our findings may be generalized to other academic environments, or to either the group private practice or solo practitioner model.

## CONCLUSIONS

The improvements typically witnessed with regard to increasing efficiency and safety over the course of the reduction mammaplasty procedural learning curve do not appear to come at the expense of esthetic outcomes, as assessed through patient-reported satisfaction metrics. This finding is particularly relevant to the reduction mammaplasty procedure, which includes both functional and esthetic components that must be considered equally when assessing surgical success.

## Figures and Tables

**Table 1:** Sample representativeness

	Responders	Nonresponders	*P*
**No. of surgeons**	**8**	**8**	
Mean experience, y*	11.2 ± 4.5	10.9 ± 4.8	NS
**No. of procedures**	**279**	**789**	
Patient mean age, y	38.7 ± 13.2	35.6 ± 12.0	<.001
Patient mean BMI	31.1 ± 6.4	31.0 ± 6.1	NS
Patient with comorbidity†			
Yes	22 (7.9%)	55 (7.0%)	NS
No	257 (92.1%)	734 (93.0%)	
Mean operative time, min	131.0 ± 34.7	135.4 ± 34.2	NS
Mean reduction volume, g	1635 ± 923	1698 ± 935	NS
Operative technique			
Wise pattern inferior pedicle	232 (83.2%)	666 (84.4%)	NS
Modified Robertson or vertical scar	47 (16.8%)	123 (15.6%)	
Liposuction			
Yes	75 (26.9%)	198 (25.1%)	NS
No	204 (73.1%)	591 (74.9%)	
Time of day			
7:30-9:59	103 (36.9%)	286 (36.2%)	
10:00-12:59	98 (35.1%)	254 (32.2%)	NS
13:00+	78 (28.0%)	249 (31.6%)	
Year of procedure			
1995-1998	44 (15.8%)	286 (36.2%)	
1999-2001	69 (24.7%)	164 (20.8%)	<.001
2002-2004	99 (35.5%)	196 (24.8%)	
2005-2007	67 (24.0%)	143 (18.1%)	
Complication‡	26 (9.3%)	54 (6.8%)	
Hematoma	5 (1.8%)	5 (0.6%)	
Necrosis	8 (2.9%)	15 (1.9%)	NS
Infection	13 (4.7%)	30 (3.8%)	
Reoperation	13 (4.7%)	19 (2.4%)	

*The length of experience variable was calculated as the number of years since the surgeon's graduation.

†Composite variable including coronary artery disease, asthma, chronic obstructive pulmonary disease, diabetes, hypertension, smoking, chronic renal insufficiency or failure, and/or cancer.

‡Composite outcome including hematoma and/or necrosis and/or infection and/or reoperation.

NS indicates nonsignificant.

**Table 2:** Univariate analyses of factors associated with satisfaction scores

		Satisfaction With Breasts (n = 277)		Satisfaction With Overall Outcome (n = 275)	
		Mean Score Value (SD)	*P*	Mean Score Value (SD)	*P*
Surgeon experience, y*	<5	65.0 ± 20.7	.776	78.8 ± 23.0	.347
	5-9	68.0 ± 21.1		83.1 ± 19.8	
	10-15	66.9 ± 21.8		81.6 ± 19.9	
	≥15	69.8 ± 20.4		86.5 ± 18.4	
Patient age, y	<26	61.1 ± 17.8	.007	84.0 ± 18.4	.947
	26-35	64.1 ± 20.3		82.1 ± 19.5	
	36-45	70.0 ± 22.9		82.0 ± 21.2	
	≥46	72.3 ± 21.0		83.3 ± 20.1	
Patient BMI	<25	68.4 ± 14.5	.787	87.4 ± 11.4	.428
	25-29	67.9 ± 19.6		82.7 ± 18.9	
	≥30	65.7 ± 23.2		79.7 ± 22.1	
Patient with comorbidity†	Yes	62.4 ± 23.5	.285	83.0 ± 19.5	.846
	No	68.0 ± 20.9		80.2 ± 24.3	
Operative time, min	<100	68.8 ± 18.3	.702	81.8 ± 18.2	.934
	100-124	69.1 ± 22.6		83.3 ± 19.6	
	125-149	65.8 ± 21.2		82.7 ± 20.7	
	≥150	66.6 ± 20.5		82.6 ± 20.6	
Reduction volume, g	<1050	68.3 ± 19.7	.440	81.5 ± 19.0	.580
	1050-1499	70.4 ± 19.6		84.7 ± 18.8	
	1500-2049	65.0 ± 24.4		83.4 ± 20.6	
	≥2050	65.8 ± 20.3		80.7 ± 21.8	
Operative technique	Wise pattern inferior pedicle	67.7 ± 20.8	.702	82.8 ± 20.0	.745
	Modified Robertson or vertical scar	66.7 ± 23.0		82.4 ± 19.2	
Liposuction	Yes	64.3 ± 20.8	.159	84.0 ± 19.0	.615
	No	68.8 ± 21.2		82.3 ± 20.2	
Time of day	7:30-9:59	67.6 ± 22.9	.786	80.1 ± 21.3	.389
	10:00-12:59	68.9 ± 19.5		84.9 ± 18.0	
	13:00+	66.2 ± 21.5		82.6 ± 20.4	
Year of procedure	1995-1998	72.1 ± 17.9	.093	84.7 ± 18.4	.521
	1999-2001	67.2 ± 22.6		84.4 ± 20.0	
	2005-2007	64.0 ± 21.5		81.1 ± 19.5	
	2002-2004	70.3 ± 20.6		82.4 ± 21.3	
Complication‡	Yes	63.8 ± 18.1	.255	76.1 ± 20.2	.047
	No	68.0 ± 21.4		83.4 ± 19.7	
Hematoma	Yes	70.8 ± 16.8	.883	92.8 ± 10.5	.274
	No	67.5 ± 21.2		82.6 ± 20.0	
Necrosis	Yes	50.2 ± 17.7	.019	64.4 ± 13.7	.004
	No	68.1 ± 21.0		83.3 ± 19.8	
Infection	Yes	71.8 ± 19.6	.447	77.6 ± 24.1	.453
	No	67.4 ± 21.2		83.0 ± 19.6	
Reoperation	Yes	63.5 ± 19.3	.303	74.1 ± 23.1	.136
	No	67.8 ± 21.2		83.2 ± 19.6	

*The length of experience variable was calculated as the number of years since the surgeon's graduation.

†Composite variable including coronary artery disease, asthma, chronic obstructive pulmonary disease, diabetes, hypertension, smoking, chronic renal insufficiency or failure, and/or cancer.

‡Composite outcome including hematoma and/or necrosis and/or infection and/or reoperation.

**Table 3:** Characteristics of survey respondents by satisfaction score quartile

Satisfaction With Breasts (n = 277)	Very Low Score (n = 70)	Low Score (n = 70)	High Score (n = 64)	Very High Score (n = 73)	*P*
Mean surgeon experience, y*	11.1 [10.1-12.2]	10.9 [9.9-11.9]	10.8 [9.5-12.0]	12.0 [10.9-13.1]	.353
Mean patient age, y	36.2 [33.5-39.0]	37.5 [34.2-40.9]	37.9 [34.2-41.6]	43.1 [40.4-45.8]	.004
Mean operative time, min	134.2 [125.9-142.5]	133.3 [124.9-141.7]	129.3 [119.3-139.3]	127.2 [120.4-133.9]	.769
Mean reduction volume, g	1740 [1523-1959]	1621 [1370-1873]	1590 [1401-1779]	1607 [1386-1827]	.441
Wise pattern inferior pedicle rate	81.4 [70.3-89.7]	82.9 [72.0-90.8]	87.5 [76.8-94.4]	80.8 [69.9-89.1]	.732
Complication rate†	4.3 [0.9-12.0]	21.4 [12.5-32.9]	7.8 [2.6-17.3]	4.1 [0.9-11.5]	.002
Necrosis rate	2.9 [0.3-9.9]	8.6 [3.2-17.7]	0.0 [0-5.6]	0.0 [0-4.9]	.003
Satisfaction With Overall Outcome (n = 275)	Very Low Score (n = 75)	Low Score (n = 43)	High Score) (n = 33)	Very High Score (n = 124)	*P*
Mean surgeon experience, y*	10.9 [9.9-11.9]	10.9 [9.4-12.4]	11.4 [10.0-12.9]	11.6 [10.8-12.4]	.639
Mean patient age, y	38.1 [35.4-40.8]	38.3 [34.3-42.3]	36.8 [31.5-42.1]	39.7 [37.3-42.2]	.571
Mean operative time, min	134.7 [126.4-143.0]	129.5 [117.3-141.7]	128.4 [117.3-139.5]	129.8 [123.9-135.7]	.635
Mean reduction volume, g	1691 [1450-1932]	1666 [1338-1993]	1402 [1180-1623]	1663 [1509-1817]	.492
Wise pattern inferior pedicle rate	81.3 [70.7-89.4]	81.4 [66.6-91.6]	87.9 [71.8-96.6]	83.1 [75.3-89.2]	.879
Complication rate†	14.7 [7.6-24.7]	9.3 [2.6-22.1]	9.1 [1.9-124.3]	5.6 [2.3-11.3]	.191
Necrosis rate	6.7 [2.2-14.9]	4.6 [0.6-15.8]	0.0 [0-10.6]	0.0 [0-2.9]	.010

*The length of experience variable was calculated as the number of years since the surgeon's graduation.

†Composite outcome including hematoma and/or necrosis and/or infection and/or reoperation.

**Table 4:** Multivariate analyses of factors associated with satisfaction scores

	Satisfaction With Breasts (n = 277)		Satisfaction With Overall Outcome (n = 275)	
	F Value	*P*	F Value	*P*
Complication (Yes/No)	0.67	0.413	3.50	0.062
Surgeon experience, y	0.05	0.820	2.46	0.118
Operative technique	0.02	0.980	0.27	0.765
Operative time, min	0.74	0.390	0.17	0.677
Patient age, y	13.31	<0.001	0.39	0.534
Reduction volume, g	0.03	0.860	0.04	0.834
